# Iron, folic acid, and vitamin D supplementation during pregnancy: Did pregnant Chilean women meet the recommendations during the COVID pandemic?

**DOI:** 10.1371/journal.pone.0293745

**Published:** 2023-11-02

**Authors:** María F. Mujica-Coopman, María Luisa Garmendia, Camila Corvalán

**Affiliations:** Institute of Nutrition and Food Technology (INTA), University of Chile, Santiago, Chile; Poznan University of Life Sciences: Uniwersytet Przyrodniczy w Poznaniu, POLAND

## Abstract

**Background:**

Antenatal micronutrient supplementation has been defined as a priority for Low-and Middle-income Countries (LMICs). However, it is also relevant to assess its performance in middle-high income countries, such as Chile, particularly given the post-pandemic food insecurity context.

**Aim:**

To assess the use (frequency and doses) of daily recommended supplementation (iron (15–30 mg), folic acid (FA) (400–800 μg/day), and vitamin (VD) (400 IU)) in a sample of Chilean pregnant women.

**Methods:**

In 1, 507 pregnant women selected from public health care registries of the Southeast area of Santiago-Chile, we collected maternal, supplement use, sociodemographic, and nutritional information at the first (<15 weeks), second (24–28 weeks), and third trimesters (32–36 weeks) of gestation by using a researcher administer online questionnaire.

**Results:**

The median (IQR) age of women was 29 (25–33) years. Pre-conceptional supplementation was rare (24%), but it reached >93% in the first trimester; thereafter supplement use decreased to 79% in the second and 84% in the third trimesters, particularly in women with lower income (p<0.05), lower education (p<0.05), and with excess weight (p<0.05). Use of iron supplements in the first trimester was rare (<21%) as well as the use of VD supplements across pregnancy (<31%). Most FA (70%) and iron (80%) supplement users, exceeded the recommended daily dose while ~40% of VD users took less than the recommended dose.

**Conclusions:**

In this sample of Chilean women, timely initiation of FA, iron, and VD supplementation was low and doses were not aligned with the recommendations. Strengthening adherence and quality of micronutrient supplementation programs delivered through public primary care could benefit particularly the most vulnerable women.

## Introduction

The prevention of micronutrient-related deficiencies such as iron deficiency anemia (IDA) and folate deficiency during pregnancy has been a priority for most Low-and Middle-income Countries (LMICs) [1–3]. Supplementation with micronutrients is the most effective strategy to prevent the adverse health consequences of micronutrient deficiency during pregnancy [4] beginning as early as possible [5]. The benefits of folic acid (FA) supplementation for improving folate concentration [4] and preventing the occurrence and reoccurrence of neural tube defects (NTDs) are well-established [1,6,7]; in turn, iron supplementation during pregnancy decreases the prevalence of anemia by 70% [8]. Recent evidence also suggests that antenatal multiple micronutrient supplementation (MMS) including multiple vitamins and minerals could further improve maternal and infant outcomes and thus, the World Health Organization (WHO) recommendations have been revised to recommend MMS for pregnant women from LMICs [5]. However, there is still debate on this topic, particularly with respect to unintended consequences that could be derived from high concentrations as well as from interaction among vitamins [9,10]. Contrary to the recommendation of iron and FA supplementation, there is no international consensus to recommend vitamin D (VD) supplementation during pregnancy [11]. Despite this, low maternal VD concentration has been associated with a higher risk of preeclampsia, gestational diabetes, and impaired bone formation and adiposity in the offspring [11,12].

Chile is a Latin-American country that has experienced important economic improvements in the last decades, being presently listed as a high-income country by the World Bank. Similarly, nutrition has rapidly evolved in the last 4–5 decades from a high prevalence of undernutrition and micronutrient deficiency to a high prevalence of obesity (78%) [13] and very low prevalence of short stature (13%) and anemia (8%) [14]. Currently, the Chilean Perinatal Guidelines recommend that all low-risk childbearing-age women should consume a daily supplement of 400–800 μg of FA starting 3 months before conception until 12 weeks after conception, a 400 IU of VD supplement through pregnancy, and an increase of 15–30 mg of iron intake through supplements starting at 16 weeks of pregnancy [15]; multiple micronutrient supplementation is recommended in women with inadequate diets.

As in several countries worldwide, given the ongoing food crises, there is concern that micronutrient deficiencies may increase particularly among vulnerable populations [16,17]. Recently, the Chilean Ministry of Health recommended as part of the national plan for micronutrient control to continue FA, iron, and VD supplementation in pregnant women to avoid micronutrient deficiencies. The Chilean public health care system (which provides care to almost 77% of the population) [18] provides free iron and FA supplements during pregnancy; however, currently, there is a lack of information on adherence to supplementation guidelines. Thereby, the objectives of the present study are 1) to assess supplemental intakes of iron, FA, and VD before pregnancy and in all trimesters of pregnancy, and 2) to identify maternal and demographic predictors of periconceptional and prenatal micronutrient supplement use in a sample of Chilean pregnant women beneficiaries of the public health system.

## Participants and methods

This study is nested within the Chilean Maternal & Infant Cohort study-II (CHiMINCs-II) [19]. Briefly, the CHiMINCs-II is a prospective cohort study that includes pregnant women and their offspring receiving prenatal care within 8 public health care centers (PHCC) in the South East Area of Santiago, Chile. Women were selected from the public health care center’s registries in the South East area of Santiago, Chile. Between March 2020 and November 2021, healthy pregnant women (≥18 years) who received prenatal care and met the eligibility criteria in any of the PHCCs were invited at their first control (<15 weeks) to participate in the CHiMINCs-II study (n = 1,954). All participants were followed across the first (<15 weeks of gestation), second (24–28 weeks of gestation), and third trimester (32–36 weeks of gestation) of pregnancy, and postnatally.

### Inclusion criteria

Pregnant women >18 years, with <15 weeks of gestation in their first prenatal control.

### Exclusion criteria

**Fig 1:**
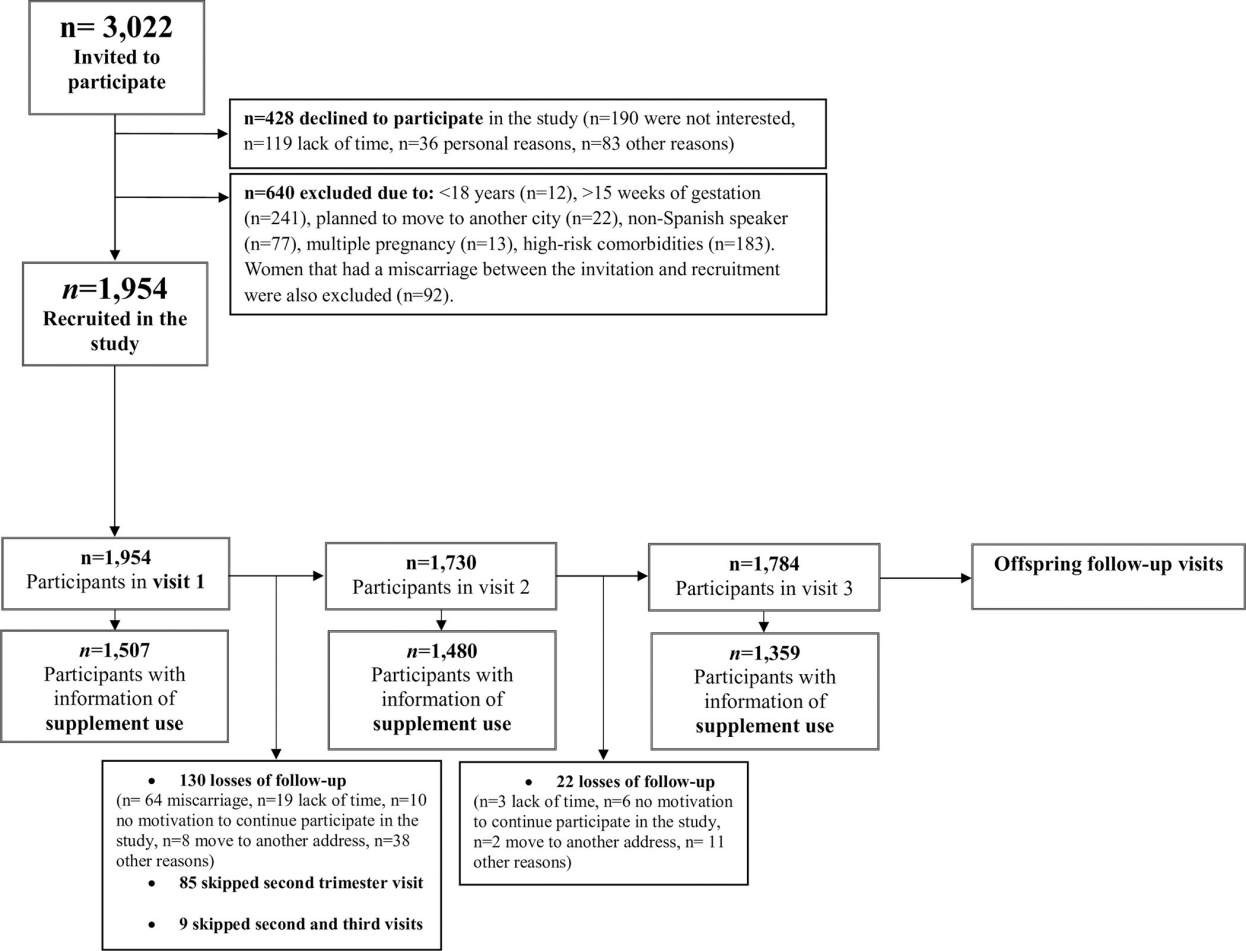
Flow chart of the participants.

Pregnant women who had planned to move to another city, and were non-Spanish speakers were excluded from the study. We also excluded pregnant women if they had multiple pregnancies, or if they had a miscarriage of the current pregnancy, or had non-compatible medical conditions. For the purposes of this study, we also excluded women with no information on supplement use (**Fig 1**).

### Ethics approval

The CHiMINCs-II study was reviewed and approved by the Institutional Review Boards of the Institute of Nutrition and Food Technology (INTA), the University of Chile, and the Southeast Metropolitan Health Service. Participants provided written or informed consent before conducting any evaluation and understood that they could withdraw from the study at any moment. Participants also provided informed consent to provide access to their clinical records.

### Sociodemographic, obstetric, and supplement use information

The research visits were conducted by phone. At the first study visit (i.e., the first trimester of pregnancy), information on maternal age (years), parity (nulliparous/multiparous), maternal education (≤12 years/ >12 years), marital status (married/non-married), total monthly income (< CLP 250.000, 297 USD; CLP 250.000–500.000, 297–598 USD; > CLP 500.000, 598 USD), pre-pregnancy weight, height, and weeks of gestation at the first study visit were collected. Also, we obtained self-reported information on anemia before pregnancy (i.e., at the first study visit). Information on alcohol consumption (yes/no), drugs (yes/no), and tobacco use (yes/no) before and during pregnancy was collected at the first and third trimester of pregnancy, respectively. We used the pre-pregnancy weight and height to calculate the pre-pregnancy body mass index (BMI). Pre-pregnancy BMI was used to classify pregnant women as normal weight (BMI <25 kg/m^2^) and excess weight (BMI ≥<25 kg/m^2^).

Information on supplement use (i.e., type of supplement) and the number of capsules consumed per day in all trimesters was collected by trained dietitians using a photographic atlas which was sent by WhatsApp to facilitate the identification of the supplement. Whether pregnant women used supplements before pregnancy was also asked at the first visit. The atlas included the most common supplements available in the market as well as those delivered free by the public health care system. We recorded whether the supplement was of one vitamin/mineral or multivitamins; although the public health care system by norm only provides iron and FA it is possible for them to provide them in a multivitamin supplement. For the micronutrient dose content, we used the information provided by the manufacturer. In the case of iron, chemical forms (ferrous sulfate, ferrous fumarate, etc.) were transformed into elemental iron, and in the case of VD, doses were all transformed into international units (IU). We categorized the daily dose of FA (i.e., ≤400 μg/day, 400-800μg, >800μg, and ≥1000μg), iron (i.e., <15mg, 15–30 mg, >30 mg, and ≥60mg/day) and VD (i.e., <400 IU, 400 IU, >400 IU) supplement according to the recommendations of the Chilean Perinatal Guidelines [15].

### Statistical analysis

The normality of the variables was tested using the Shapiro-Wilk test. Continuous variables were expressed as mean±standard deviation (SD) or median (interquartile range). Categorical variables were expressed as percentages (%). A Wilcoxon rank sum test was used to compare maternal age and pre-pregnancy BMI by supplement users and non-users of supplements before pregnancy and in each trimester. Maternal education, civil status, total monthly income, BMI categories, parity, smoking habit, alcohol consumption, drugs use, and history of anemia before pregnancy were compared among users and non-users using a Chi-square test. Stepwise logistic regressions were used to estimate which maternal and demographic characteristics were the best predictors of the use of supplements before pregnancy and in each trimester. Variables were retained in the model if *P*<0.05. All the analyses were conducted using STATA SE 17.

## Results

### Participant characteristics

We recruited 1,507 women for the study. The median and interquartile range (IQR) age of women was 29 (25–33) years, 19.5% were married and 69% had ≤ 12 years of education. Of the total sample, 74% had excess weight (i.e., BMI ≥25 kg/m^2^), 15% had a history of anemia before pregnancy, and 24% of women used micronutrient supplements before pregnancy while this number increased to 94%, 79%, and 84% at the first, second, and third trimesters of pregnancy. Regarding lifestyle habits, non-supplement users before pregnancy had lower education, income, had more excess weight, and were more smokers compared to supplement users (**Table 1**). During pregnancy, <%5 declared using alcohol, tobacco, or drugs.

**Table 1:** Maternal and demographic characteristics of pregnant women participating in the CHIMINCs-II study among supplement users and non-users before pregnancy (n = 1,506) ^a^.

	Users (n = 360)	Non-Users (n = 1,146)	*P* value^b^
Age (years) (n = 1506)	30 (27–34)	28 (24–33)	<0.001
Marital status, %			<0.001
*Married*	28.1 (101)	16.9 (194)	
*Non-married*	71.9 (259)	83.1 (952)	
Monthly income, CLP (USD), % (n = 1,315)			<0.001
*<$250*.*000 (297)*	8.54 (n = 28)	11.5 (n = 113)	
*$250*.*000–500*.*000 (297–598)*	26.5 (n = 87)	41.0 (n = 405)	
*>$500*.*000 (598)*	64.9 (n = 213)	47.5 (n = 469)	
Maternal education, %			<0.001
*≤12 years*	52.2 (n = 188)	74.2 (n = 850)	
*>12 years*	47.8 (n = 172)	25.8 (n = 296)	
Pre-pregnancy BMI (n = 1,413)	27.4 (24.0–32.2)	28.6 (25.0–33.2)	0.005
BMI categories, %			0.018
*Normal weight*	32.0 (n = 108)	25.4 (n = 278)	
*Excess weight*	68.0 (n = 230)	74.6 (n = 815)	
History of anemia before pregnancy, %			<0.001
*Yes*	20.3 (n = 73)	12.8 (n = 147)	
*No*	79.7 (n = 287)	87.2 (n = 999)	
Parity, %			0.007
*0–1 living children*	77.2 (n = 278)	69.3 (n = 794)	
*2–3 living children* *> 3 living children*	20.8 (n = 75) 1.94 (n = 7)	29.2 (n = 335) 1.48 (n = 17)	
Smoking before pregnancy, %			0.017
*Yes*	39.4 (n = 143)	46.6 (n = 534)	
*No*	60.6 (n = 220)	53.4 (n = 612)	
Alcohol consumption before pregnancy, %			0.856
*Yes*	60.8 (n = 219)	60.3 (n = 691)	
*No*	39.2 (n = 141)	39.7 (n = 455)	
Drugs use before pregnancy, %			0.174
*Yes*	16.7 (n = 60)	19.9 (n = 228)	
*No*	83.3 (n = 300)	80.1 (n = 918)	

^a^Continuous variables are expressed as median (IQR). Categorical variables are expressed as percentages.

^b^Wilcoxon rank-sum test and chi-square test to compare continuous variables and percentages, respectively, among users and non-users before pregnancy.

### Maternal and demographic differences among users and non-users

Except in the 1^st^ trimester, we observed that women who were non-supplement users had significantly lower income and educational levels and more excess weight (in all trimesters) compared to users (**Tables 1 and 2**). Also, non-users had more living children (i.e., >1 child) than users, and in the case of pre-pregnancy and in the 2^nd^ trimester, non-users were younger than users (**Table 2**). In the 1^st^ trimester, only having a lower income was significantly related to the use of supplements. In the multiple variable analyses, most of the same variables remained significant (**S1–S4 Tables**).

**Table 2:** Maternal and demographic characteristics of pregnant women participating in the CHIMINCs-II study among supplement users and non-users in each trimester of pregnancy^a^.

	1^st^ trimester Users (n = 1411)	1^er^ trimester Non-Users (n = 96)	2^nd^ trimester Users (n = 1170)	2^nd^ trimester Non-Users (n = 310)	3^rd^ trimester Users (n = 1135)	3rd trimester Non-Users (n = 224)	*P* ^*b*^	*P* ^*c*^	*P* ^*d*^
Age (years)	29 (24–33)	28 (25–31)	29 (25–33)	27 (23–32)	29 (24–33)	28 (24–33)	0.367	0.003	0.570
Marital status, %							0.208	0.263	0.715
*Married*	19.8 (n = 280)	14.6 (n = 14)	20.9 (n = 245)	18.1 (n = 56)	20.3 (n = 230)	19.2 (n = 43)			
*Non-married*	80.2 (n = 1131)	85.4 (n = 82)	79.1 (n = 925)	81.9 (n = 254)	79.7 (n = 905)	80.8 (n = 181)			
Monthly income, CLP (USD)							0.002	<0.001	0.009
*<$250*.*000 (297)*	10.2 (n = 126)	20.0 (n = 15)	11.3 (n = 117)	14.0 (n = 38)	12.3 (n = 123)	12.5 (n = 25)			
*$250*.*000–500*.*000 (297–598)*	36.9 (n = 454)	45.3 (n = 34)	34.5 (n = 355)	52.6 (n = 143)	36.6 (n = 367)	47.5 (n = 95)			
*>$500*.*000 (598)*	52.9 (n = 652)	34.7 (n = 36)	54.3 (n = 561)	33.4 (n = 91)	51.2 (n = 512)	40.0 (n = 80)			
Maternal education, %							0.746	<0.001	<0.001
*< = 12 years*	68.9 (n = 970)	70.5 (n = 67)	64.8(n = 758)	83.6 (n = 259)	66.2 (n = 751)	79.9 (n = 179)			
*>12 years*	31.1 (n = 437)	29.5 (n = 28)	35.2 (n = 412)	16.4 (n = 51)	33.8 (n = 384)	20.1 (n = 45)			
Pre-gestational BMI (kg/m^2^)	28.3 (24.7–32.9)	29.1 (25.6–34.6)	28.2 (24.5–32.8)	28.6 (25.7–33.8)	27.9 (24.5–32.5)	29.6 (26.1–34.1)	0.163	0.023	0.001
Pregestational BMI categories, %							0.037	0.003	0.002
*Normal weight*	26.3 (n = 371)	16.7 (n = 16)	29.2 (n = 336)	20.7 (n = 63)	29.0 (n = 325)	18.8 (n = 42)			
*Excess weight*	73.7 (n = 1040)	83.3 (n = 80)	70.8 (n = 815)	79.3 (n = 242)	71.0 (n = 794)	81.2 (n = 81)			
History of anemia before pregnancy, %							0.997	0.306	0.230
*Yes*	14.6 (n = 206)	14.6 (n = 14)	14.4 (n = 169)	16.8 (n = 52)	15.1 (n = 172)	12.0 (n = 27)			
*No*	85.4 (n = 1205)	85.4 (n = 82)	85.6 (n = 1001)	83.2 (n = 258)	84.9 (n = 963)	88.0 (n = 197)			
Parity, %							0.230	0.004	0.001
*0–1 living children*	71.7 (n = 1012)	63.5 (n = 61)	73.6 (n = 861)	64.5 (n = 200)	72.8 (n = 826)	61.2 (n = 137)			
*2–3 living children* *> 3 living children*	26.7 (n = 377) 1.56 (n = 22)	34.4 (n = 33) 2.08 (n = 2)	25.1 (n = 294) 1.28 (n = 15)	32.9 (n = 102) 2.58 (n = 8)	26.0 (n = 295) 1.23 (n = 14)	35.7 (n = 80) 3.12 (n = 7)			
Smoking before pregnancy, %							0.138	0.381	0.426
*Yes*	44.3 (n = 625)	52.1 (n = 50)	39.8 (n = 704)	37.1 (n = 115)	38.2 (n = 434)	41.1 (n = 92)			
*No*	55.7 (n = 786)	47.9 (n = 46)	60.2 (n = 466)	62.9 (n = 195)	61.8 (n = 701)	58.9 (n = 132)			
Alcohol consumption before pregnancy, %							0.652	0.015	0.079
*Yes*	60.2 (n = 849)	62.5 (n = 60)	55.8 (n = 653)	48.1 (n = 149)	55.1 (n = 625)	48.7 (n = 109)			
*No*	39.8 (n = 562)	37.5 (n = 36)	44.2 (n = 517)	51.9 (n = 161)	44.9 (n = 510)	51.3 (n = 115)			
Drugs use before pregnancy, %							0.652	0.391	0.991
*Yes*	19.0 (n = 267)	20.8 (n = 20)	18.8 (n = 196)	21.2 (n = 54)	20.1 (n = 200)	20.1 (n = 38)			
*No*	81.0 (n = 1141)	79.2 (n = 76)	81.2 (n = 846)	78.8 (n = 201)	79.9 (n = 793)	79.9 (n = 151)			

^a^Continuous variables are expressed as median (IQR). Categorical variables are expressed as percentages.

^b,c,d^Wilcoxon rank sum test, and chi-square test to compare continuous variables and percentages, respectively, among users and non-users at each trimester.

### Iron supplement use

For iron supplementation, the use increased with pregnancy from <20% in the first trimester to >70% in the second and third trimesters. In users, iron daily consumption from supplements ranged between 2–457 mg elemental iron with a median ≥ 40 mg elemental iron in all trimesters. Not meeting the recommendation of iron supplementation (i.e., 15–30 mg iron) was uncommon, being <13% in all trimesters; conversely, receiving a dose >30 mg was very common (>79%) (**Table 3**). Use of generic brands of iron (i.e., the one delivered by the PHCC) was infrequent (8% in the first trimester) although it slightly increased in the later trimesters (29% in the second and 21% in the third trimester). Iron was mostly consumed as part of multivitamins (54% in the first, 67% in the second, and 77% in the third trimester).

**Table 3:** Consumption of micronutrient supplements according to the limits of the perinatal guidelines by trimester in participants of the CHiMINCs-II cohort study^a^.

	1^er^ trimestre (n = 1,411)	2^nd^ trimestre (n = 1,170)	3^rd^ trimestre (n = 1,135)
**Use of micronutrient supplementation containing FA**	93.2%	68.6%	78.3%
Median (IQR) consumption of folic acid (μg)	1000 (1000–1000)	2000 (800–3000)	2000 (1000–3000)
Consumption of a daily dose of FA <400 μg/day	0.46%	2.15%	1.72%
Consumption of a daily dose of FA 400–800 μg/day^2^ Consumption of a daily dose of FA >800 μg/day	4.83% 1.46%	24.7% 1.77%	18.9% 1.95%
Consumption of a daily dose of FA ≥1000 μg/day	93.3%	71.4%	77.4%
**Use of micronutrient supplementation containing iron**	20.0%	91.5%	93.3%
Median (IQR) consumption of iron (mg)	40 (40–66)	60 (40–109)	100 (40–109)
Consumption of a daily dose of iron <15 mg/day	12.4%	7.94%	6.61%
Consumption of a daily dose of iron 15–30 mg/day^2^	7.80%	7.47%	5.48%
Consumption of a daily dose of iron >30 mg/day	36.5%	30.4%	26.6%
Consumption of a daily dose of iron ≥60 mg/day	43.3%	54.2%	61.3%
**Use of micronutrient supplementation containing VD**	7.81%	30.0%	25.3%
Median (IQR) consumption of vitamin D (IU)	400 (176–500)	400 (176–576)	400 (176–676)
Consumption of a daily dose of vitamin D <400IU/day	35.5%	35.8%	35.5%
Consumption of a daily dose of vitamin D = 400IU/day^2^	24.6%	20.3%	18.8%
Consumption of a daily dose of vitamin D >400 IU/day	40.0%	43.9%	45.7%

^a^Median and IQR. IQR = Interquartile range.

^2^Recommended dose by the National Perinatal Guidelines. FA, folic acid; VD, vitamin D.

### FA supplement use

Consumption of FA supplements was high (87% in the 1^st^ trimester, 55% in the 2^nd^ trimester, and 67% in the 3^rd^ trimester), except pre-pregnancy (22%). Daily FA consumption from supplements ranged from 71–12000 μg with a median of 1000 μg FA in the first and 2000 μg FA in the second and third trimesters. Consumption below recommended daily dose was infrequent (<5%) while consumption of high doses were frequent: >70% consumed >800 μg/day, and ~70% exceeded the tolerable upper intake level (UL) (i.e., 1000μg of FA/ day) in all trimesters of pregnancy (**Table 3**). In the first trimester, most women (>70%) consumed the generic brand of FA (i.e., which is likely the one delivered by the public health care system). In contrast, <5% of women use the generic brand of FA supplementation in the second and third trimesters. Consistently, 95.3%, 8.72%, and 5.50% of women consumed FA alone in the first, second, and third trimesters, respectively.

### VD supplement use

In contrast with FA and iron supplementation, only 7% of women took VD supplements in the first trimester, and ~30% of the participants consumed VD supplementation in the second and third trimesters. The daily dose of VD supplements ranged from 50–1600 IU. Of users, <70% of women met or exceeded the dose of VD recommended in all trimesters of pregnancy (i.e., 400 IU) (**Table 3**).

## Discussion

Prevention of micronutrient deficiency in critical developmental windows such as pregnancy has been a priority in several countries. In this context, supplementation programs are a key strategy to ensure an adequate maternal nutritional status. In Chile, the current national perinatal guidelines state that all women capable of being pregnant should consume 400–800 ug of FA starting 3 months before conception until 12 weeks of pregnancy. In addition, daily consumption of 15–30 mg of iron and a supplement of 400 IU of VD is recommended during pregnancy.

The present prospective cohort study in a sample of Chilean pregnant women beneficiaries of the public health system showed that only 24% of pregnant women took supplements before pregnancy and most of them were only FA. In contrast, we found that almost all women consumed micronutrient supplementation in the first trimester (94%). The adherence to FA supplementation was high in the first trimester (i.e., 93%) which is higher than the 60% adherence reported in Brazilian pregnant women [20], but similar to the adherence to FA supplementation in Canadian pregnant women (>95%) [21,22]. Conversely to the situation observed with FA, we found that only 20% of women took iron in the first trimester but this figure reached above 90% during the second and third trimesters which is much higher than what has been described in other countries [23,24]. Low use in the first trimester might be explained because current national guidelines advise increasing iron use since week 16, although WHO recommendations suggest starting supplementation as early as possible. Overall, there is still controversy on whether iron supplements will provide additional benefits in the iron-sufficient population, for example, the US Preventive Services Task Force concluded that there was insufficient evidence to recommend its routine use in iron-sufficient pregnant women [25]. Thus, recommendations might depend on the underlying nutritional status of pregnant women and are particularly relevant in the context of food insecurity.

We also observed that adherence to the recommendation for supplements containing VD was low (~20%) throughout pregnancy. A recent study in Mexican pregnant women showed similar results; <10% of women met the recommended dose of VD supplementation during pregnancy [26]. Given the prevalence of vitamin D inadequacy in women 15-49y (84%) [13], obesity (>35%) [13], and gestational diabetes (~15%) [27] in Chilean pregnant women, efforts should be made to improve compliance with VD supplementation [28,29]. Recently, VD fortification of dairy products and wheat flour has been approved in Chile, which should also contribute to improving VD status in pregnant women. Moreover, doses of VD supplementation >1000 IU during pregnancy have shown to significantly improve VD concentration [30,31] and bone health in the offspring [32], with no adverse health effects.

Differences in the adherence to micronutrient supplementation during pregnancy may be explained by several factors, such as access to the prenatal health care system, timely counseling related to the benefits of supplement use, maternal educational level, and income among others [21,33]. Our findings indicated that non-supplement user women were poorer, less educated, and had more excess weight than the users before pregnancy and in the second and third trimesters. These results are worrisome because low SES women might be exposed to a number of other perinatal risk factors that may have a synergic adverse effect on maternal and infant health outcomes [16].

Regarding the provision of supplements, we observed that >70% of pregnant women used generic brands of FA and <30% iron supplements which are the ones delivered by the public health care system. Therefore, women with a higher purchasing capacity and knowledge about the benefits of adequate micronutrient status during pregnancy [34] might be using out-of-pocket money to access multiple micronutrients supplements.

Regarding the supplement dose used by pregnant women, >70% of users exceeded the daily FA recommended dose (i.e., 400–800 μg), with >70% using a dose higher than the UL for FA. A recent study in Canadian pregnant women showed that marketed and prescribed FA supplements exceeded more than 2.5 times (i.e., ≥ 1000 μg of FA) the recommended daily dose for low-risk women (i.e., 400 μg FA). Despite that evidence from human studies is inconclusive regarding the potentially harmful health effects of the use of high doses of FA supplementation [35], it is well-known that doses of FA ≥1000μg alter one-carbon metabolism, which is critical for cellular proliferation and differentiation [36]. Furthermore, findings of a randomized controlled trial in Irish pregnant women showed that the extended use (i.e., during the second and third trimesters) of 400 μg of FA supplementation affected the methylome of brain-related genes in the offspring [37]. Whether the use of FA supplementation after the first trimester or the use of higher doses provides further positive benefits for the mother-dyads also needs to be confirmed.

For iron supplementation, our study showed that >40% of pregnant women using supplements had an elemental iron dose ≥of 60 mg, with a median ≥40mg each trimester. Iron needs during pregnancy are significantly high to meet metabolic maternal and fetal demands [38]. Doses of iron supplementation ≥60 mg are recommended for populations with a prevalence of iron deficiency anemia (IDA) >40% [11]. For iron supplementation, evidence of clinical trials in European pregnant women showed that doses of iron supplementation >40mg have no further benefits in the prevention of IDA [39,40]. Currently, there is a lack of information regarding the prevalence of IDA in Chilean pregnant women. However, the latest National Health Survey indicated that 5% of women have anemia. Therefore, it is likely that in this sample of pregnant women an iron overload may occur, which is alarming because iron excess may cause hypertensive disorders during pregnancy, maternal prooxidative status, mineral malabsorption [41], and a higher risk of low birth weight, and a pro-oxidative status [11].

The strengths of the present study are the inclusion of a sample of pregnant women that were followed longitudinally across all trimesters of pregnancy. In addition, the collection of supplement use information using photos and brands present in the Chilean market or delivered by the public health care system provides more accurate information on the dose and type of supplement used. However, our study also has some limitations. The use of supplements was self-reported; however, data was collected contemporary to the behavior, thus, decreasing memory bias. Also, we did not have information on clinical outcomes of micronutrient deficiencies such as anemia; but the prevalence of anemia among women of childbearing age is around 8% in Chile [14], and even for the treatment of anemia, mean doses of iron supplementation seem high. Finally, our sample is non-representative, therefore our results cannot be extrapolated to other populations such as women receiving private health care. Nonetheless, the public system provides care to most of the country (~77%) [18]. Furthermore, we collected supplement use information during the COVID-19 pandemic which may affect the attendance at prenatal care appointments and the use of free-delivered iron and FA supplements during pregnancy, although this effect has been described to be small [42].

In conclusion, in this sample of Chilean pregnant women, we observe that the timely initiation of FA, iron and VD supplementation was low, especially in vulnerable women. Additionally, a wide range of FA, VD, and iron doses are used by pregnant women which are not aligned with the national and international guidelines that may cause an excess of FA and iron consumption. Given the current food crisis, we believe these results indicate that further efforts should be made to improve adherence and quality of supplementation programs even in high-income countries such as Chile. Improving micronutrient supplement delivery will have short and long-term positive consequences.

## Supporting information

S1 Table Maternal and demographic predictors of non-supplement use before pregnancy in pregnant women participating in the CHIMINCs-II study. (DOCX)

S2 Table Maternal and demographic predictors of non-supplement use in the first trimester in pregnant women participating in the CHIMINCs-II study. (DOCX)

S3 Table Maternal and demographic predictors of non-supplement use in the second trimester in pregnant women participating in the CHIMINCs-II study. (DOCX)

S4 Table Maternal and demographic predictors of non-supplement use in the third trimester in pregnant women participating in the CHIMINCs-II study. (DOCX)
