# Supplementary material for: Iron, folic acid, and vitamin D supplementation during pregnancy: Did pregnant Chilean women meet the recommendations during the COVID pandemic?
Source: PLoS One. 2023 Nov 2;18(11):e0293745. doi: 10.1371/journal.pone.0293745 (PMC10621940; doi:10.1371/journal.pone.0293745)
Supplement: S1 Table — (DOCX) [file pone.0293745.s001.docx]

**Supplementary Table 1. Maternal and demographic predictors of non-supplement use before pregnancy in pregnant women participating in the CHIMINCs-II study**

|  | **OR** | **SE** | **CI (95%)** | ***P* value^a^** |
| --- | --- | --- | --- | --- |
| Excess weight | 1.488 | 0.213 | 1.123-1.970 | 0.006 |
| History of anemia before pregnancy | 0.579 | 0.098 | 0.415-0.808 | 0.001 |
| Non-married | 1.562 | 0.243 | 1.152-2.118 | 0.004 |
| >12 years | 0.414 | 0.055 | 0.320-0.538 | <0.001 |
| Age | 0.954 | 0.011 | 0.932-0.977 | <0.001 |

^a^Adjusted logistic model
