# Supplementary material for: Iron, folic acid, and vitamin D supplementation during pregnancy: Did pregnant Chilean women meet the recommendations during the COVID pandemic?
Source: PLoS One. 2023 Nov 2;18(11):e0293745. doi: 10.1371/journal.pone.0293745 (PMC10621940; doi:10.1371/journal.pone.0293745)
Supplement: S2 Table — (DOCX) [file pone.0293745.s002.docx]

**Supplementary Table 2. Maternal and demographic predictors of non-supplement use at the first trimester in pregnant women participating in the CHIMINCs-II study**

|  | **OR** | **SE** | **CI (95%)** | ***P* value^a^** |
| --- | --- | --- | --- | --- |
| ≥ 500.000 CLP | 0.335 | 0.113 | 0.173-0.650 | 0.001 |

^a^Adjusted logistic model
