# Supplementary material for: Iron, folic acid, and vitamin D supplementation during pregnancy: Did pregnant Chilean women meet the recommendations during the COVID pandemic?
Source: PLoS One. 2023 Nov 2;18(11):e0293745. doi: 10.1371/journal.pone.0293745 (PMC10621940; doi:10.1371/journal.pone.0293745)
Supplement: S3 Table — (DOCX) [file pone.0293745.s003.docx]

**Supplementary Table 3. Maternal and demographic predictors of non-supplement use at the second trimester in pregnant women participating in the CHIMINCs-II study**

|  | **OR** | **SE** | **CI (95%)** | ***P* value^a^** |
| --- | --- | --- | --- | --- |
| Excess weight | 1.697 | 0.300 | 1.199-2.400 | 0.003 |
| Age | 0.972 | 0.013 | 0.947-0.997 | 0.026 |
| >12 years | 0.497 | 0.092 | 0.346-0.715 | <0.001 |

**^a^**Adjusted logistic model
