# Supplementary material for: Iron, folic acid, and vitamin D supplementation during pregnancy: Did pregnant Chilean women meet the recommendations during the COVID pandemic?
Source: PLoS One. 2023 Nov 2;18(11):e0293745. doi: 10.1371/journal.pone.0293745 (PMC10621940; doi:10.1371/journal.pone.0293745)
Supplement: S4 Table — (DOCX) [file pone.0293745.s004.docx]

**Supplementary Table 4. Maternal and demographic predictors of non-supplement use at the third trimester in pregnant women participating in the CHIMINCs-II study**

|  | **OR** | **SE** | **CI (95%)** | ***P* value^a^** |
| --- | --- | --- | --- | --- |
| Excess weight | 1.704 | 0.339 | 1.155-2.517 | 0.007 |
| >12 years | 0.532 | 0.106 | 0.359-0.789 | 0.002 |

**^a^**Adjusted logistic model
